# Supplementary material for: Comprehensive Time-Course Effects of Combined Training on Hypertensive Older Adults: A Randomized Control Trial
Source: Int J Environ Res Public Health. 2022 Sep 4;19(17):11042. doi: 10.3390/ijerph191711042 (PMC9518134; doi:10.3390/ijerph191711042)
Supplement: Supplementary file 1 [file ijerph-19-11042-s001.zip › ijerph-1829102-supplementary.pdf]

Supplementary material

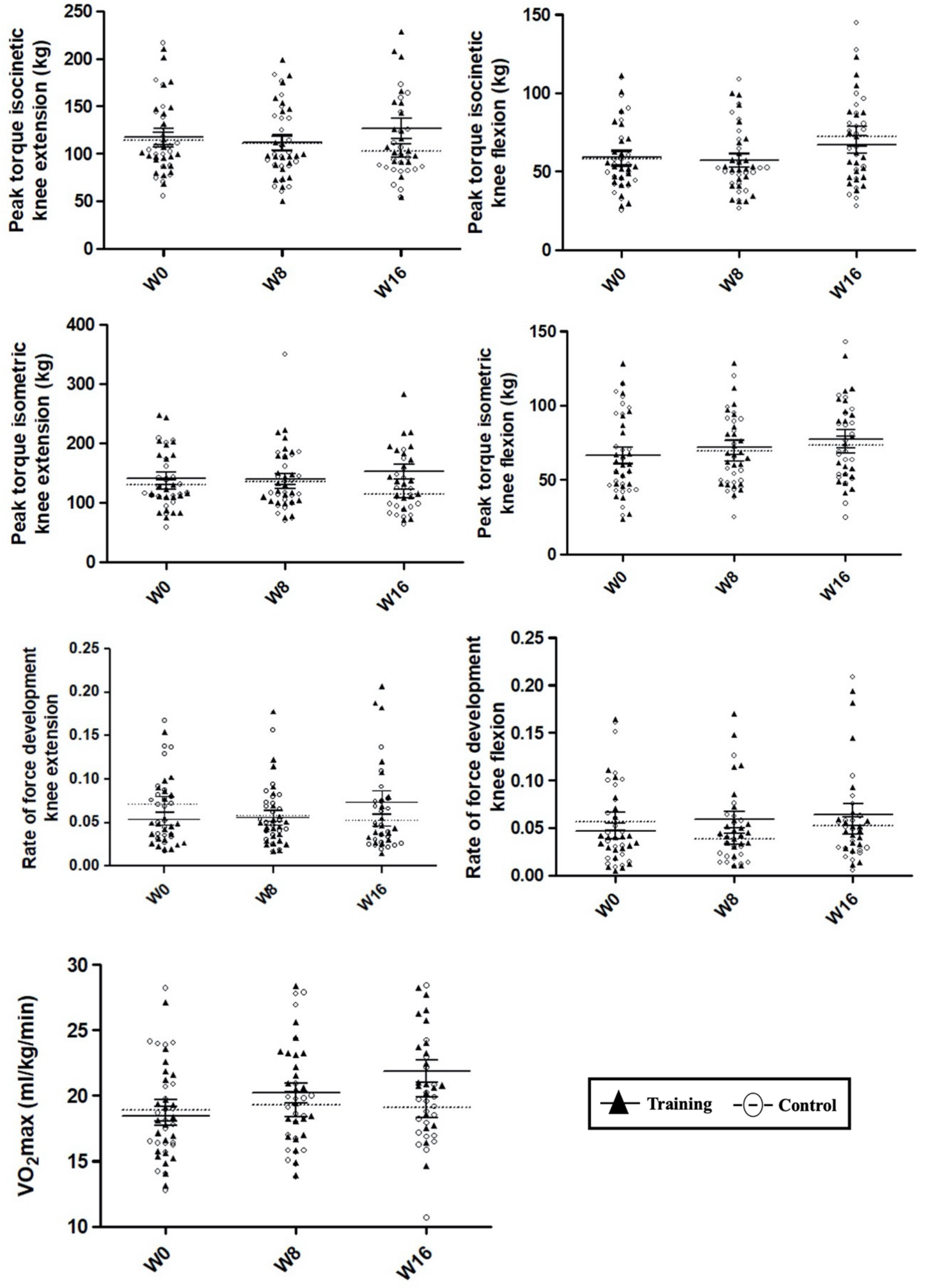

Figure S1a. Individual data, median and interquartile range for Physical fitness: Muscle strength and maximum oxygen consumption.

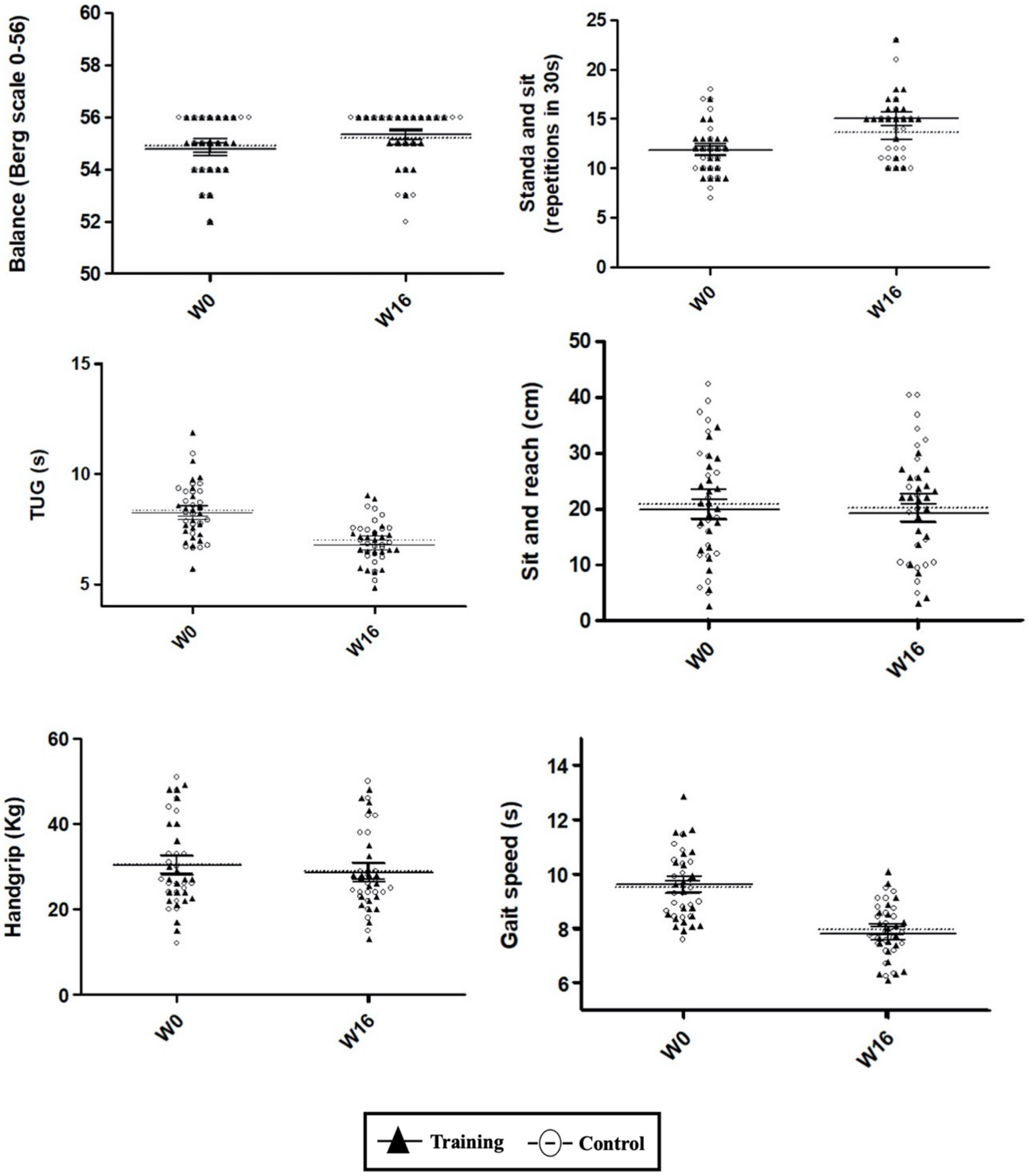

Figure S1b. Individual data, median and interquartile range for Physical fitness: Functionality.

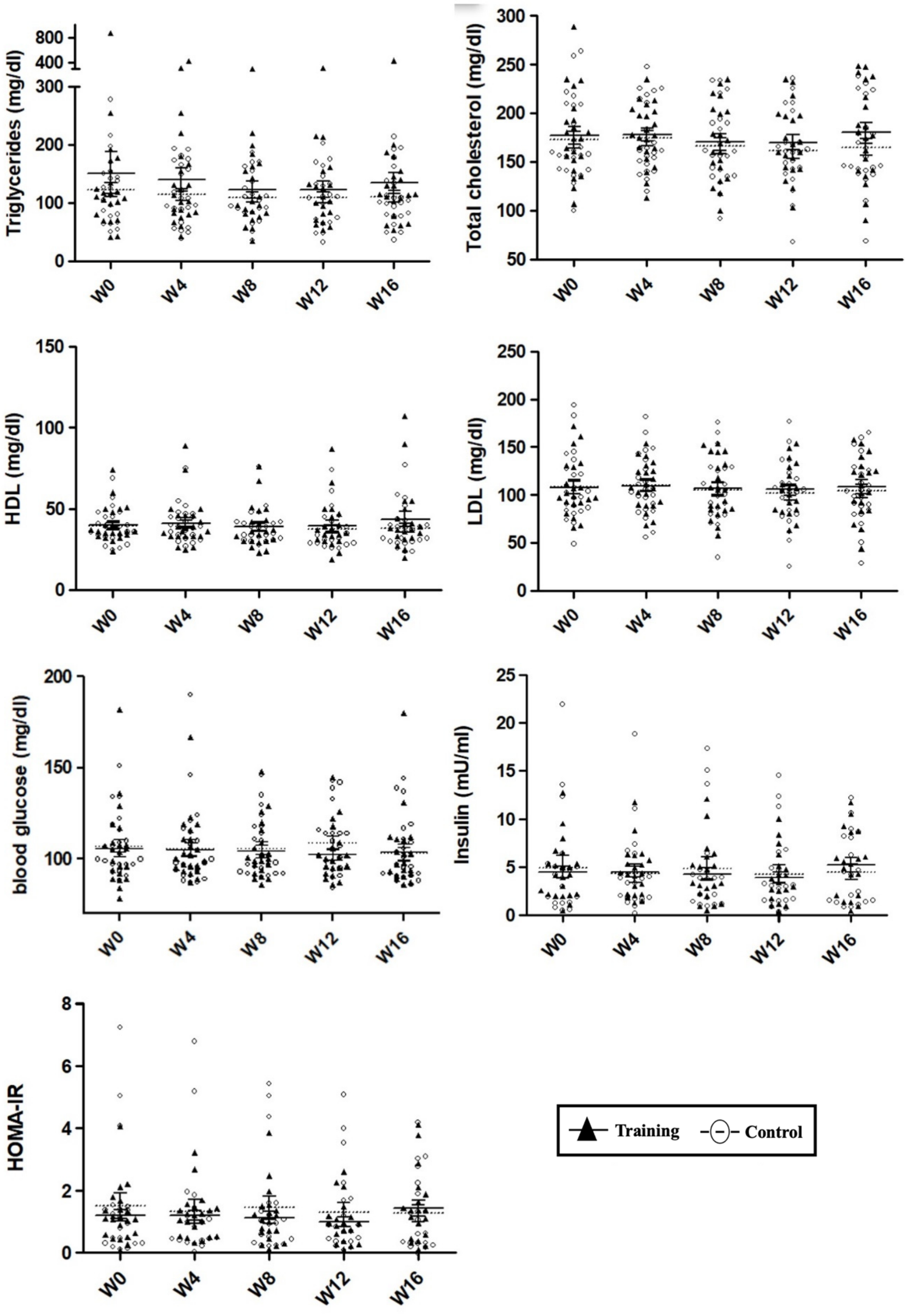

Figure S2a. Individual data, median and interquartile range for Blood markers: Glycemic and Lipidic profile.

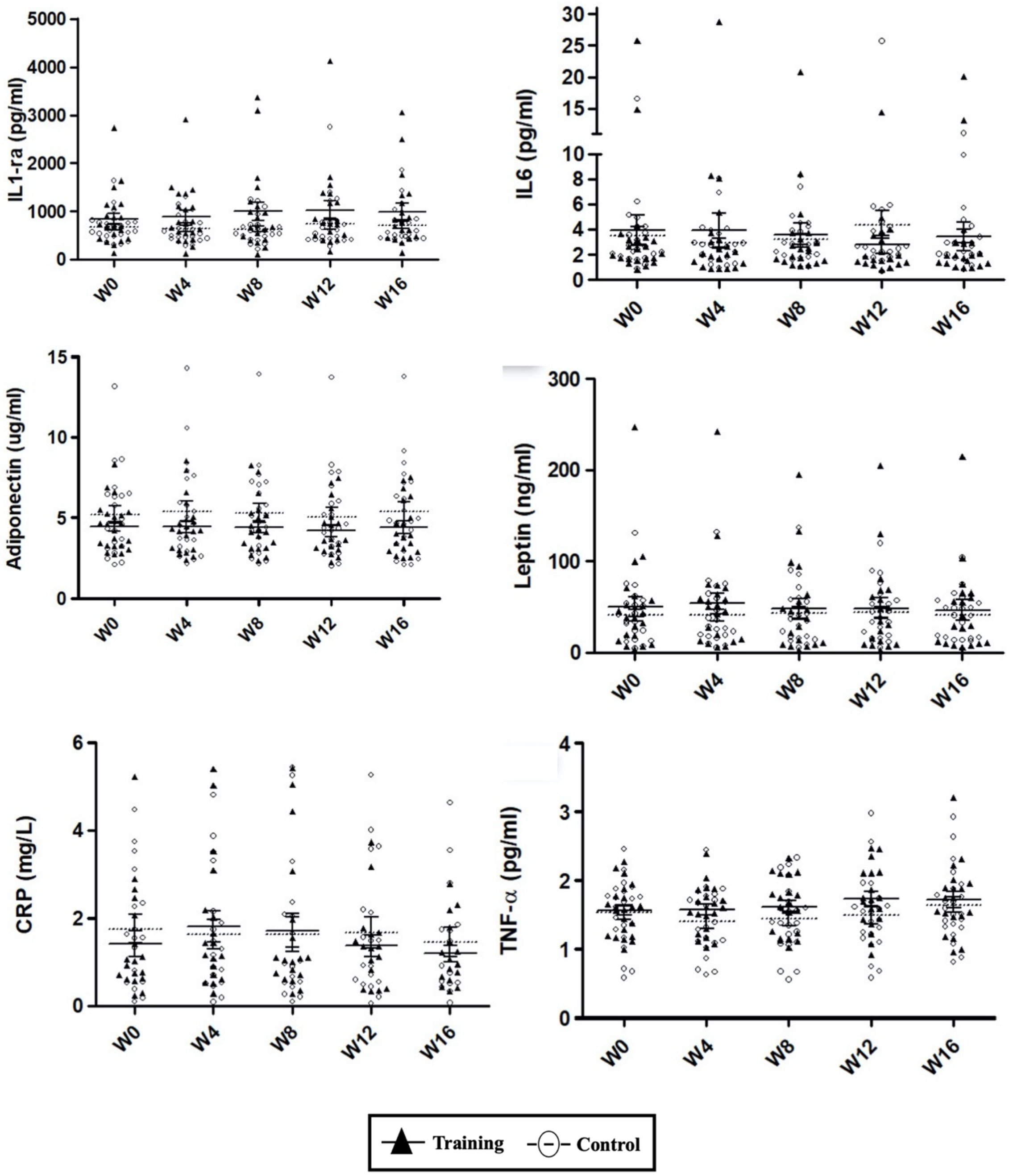

Figure S2b. Individual data, median and interquartile range for Blood markers: Inflammatory profile.

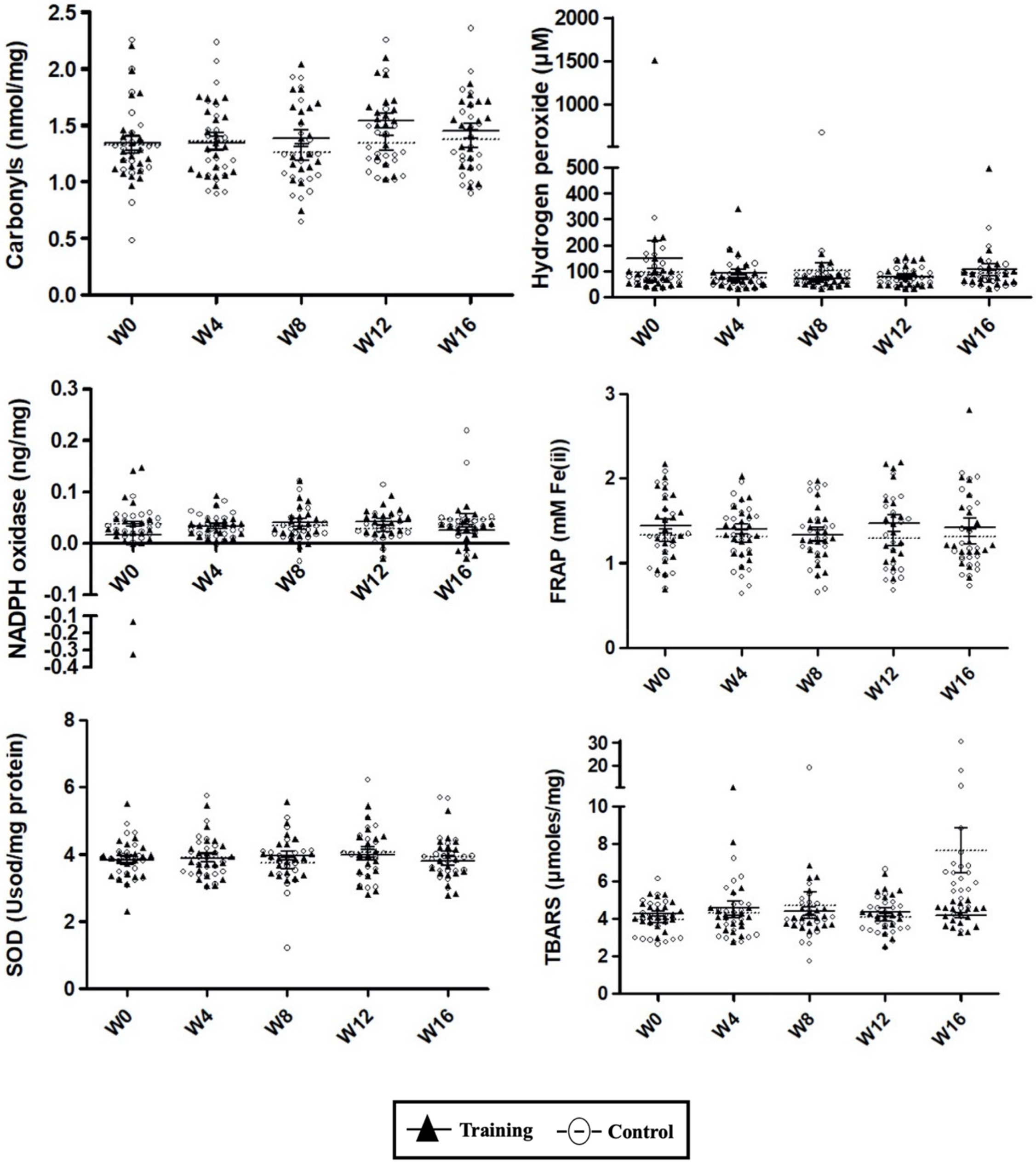

Figure S2c. Individual data, median and interquartile range for Blood markers: Oxidative stress profile.

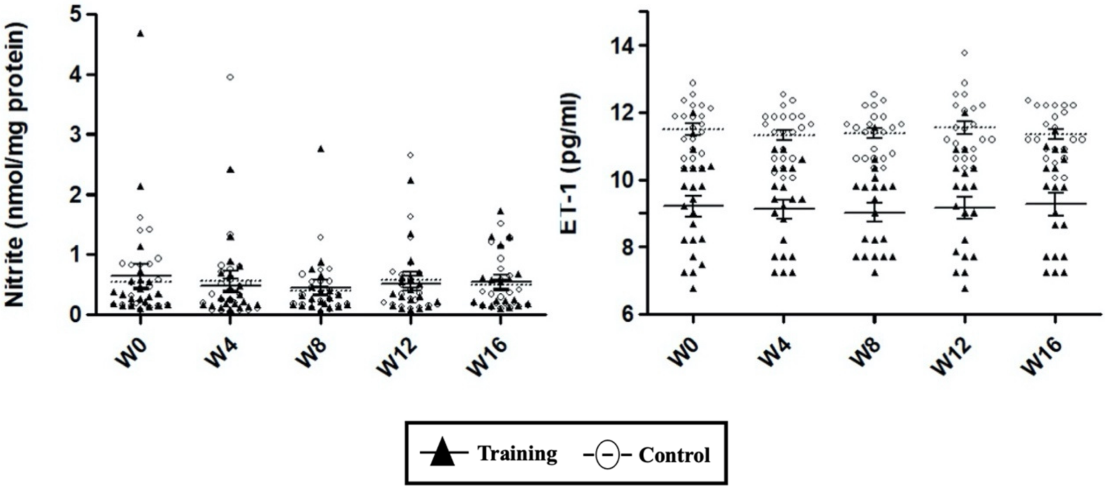

Figure S2d. Individual data, median and interquartile range for Blood markers: vasoactive substances.

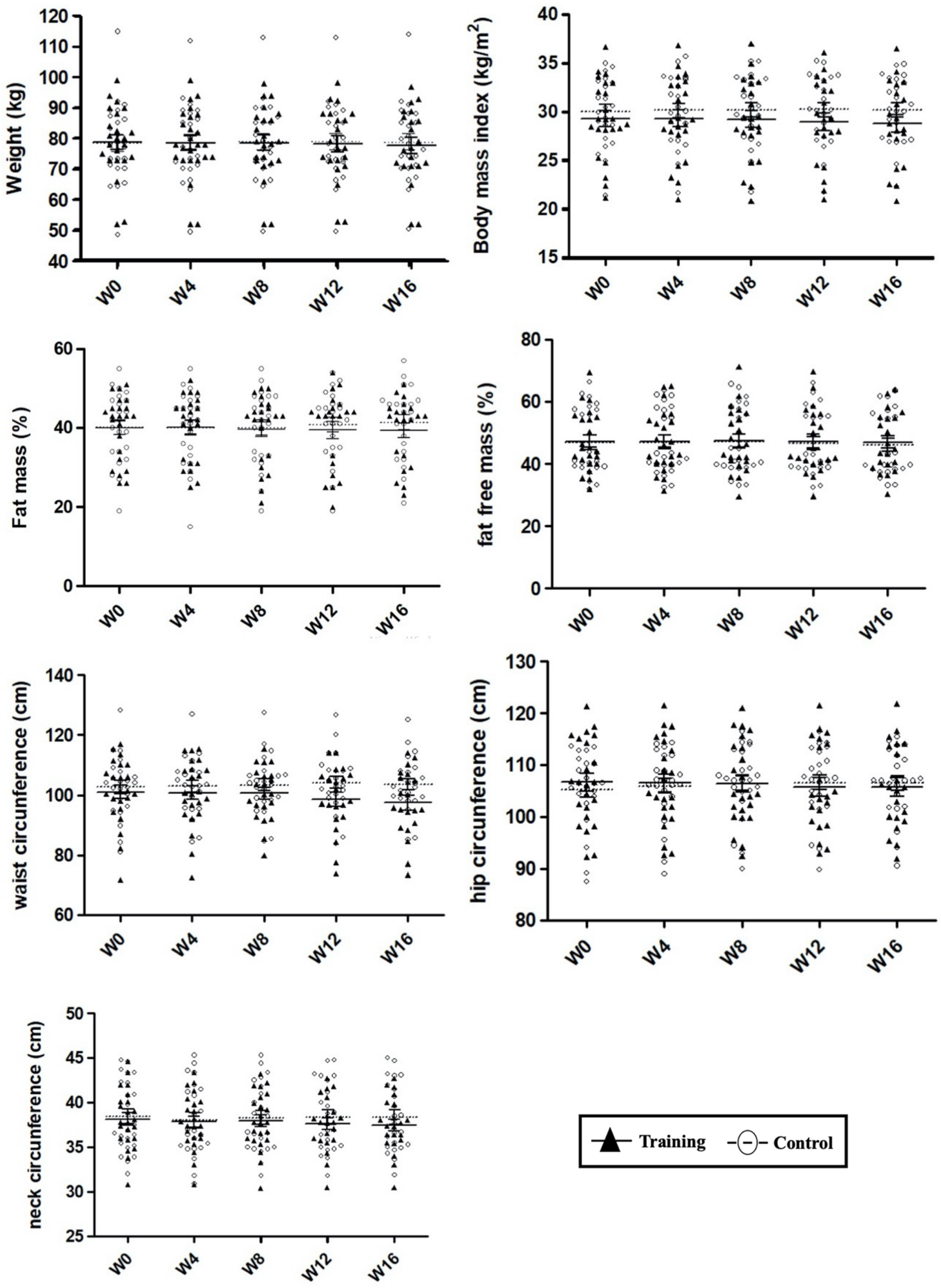

Figure S3a. Individual data, median and interquartile range for Cardiometabolic profile: Body composition.

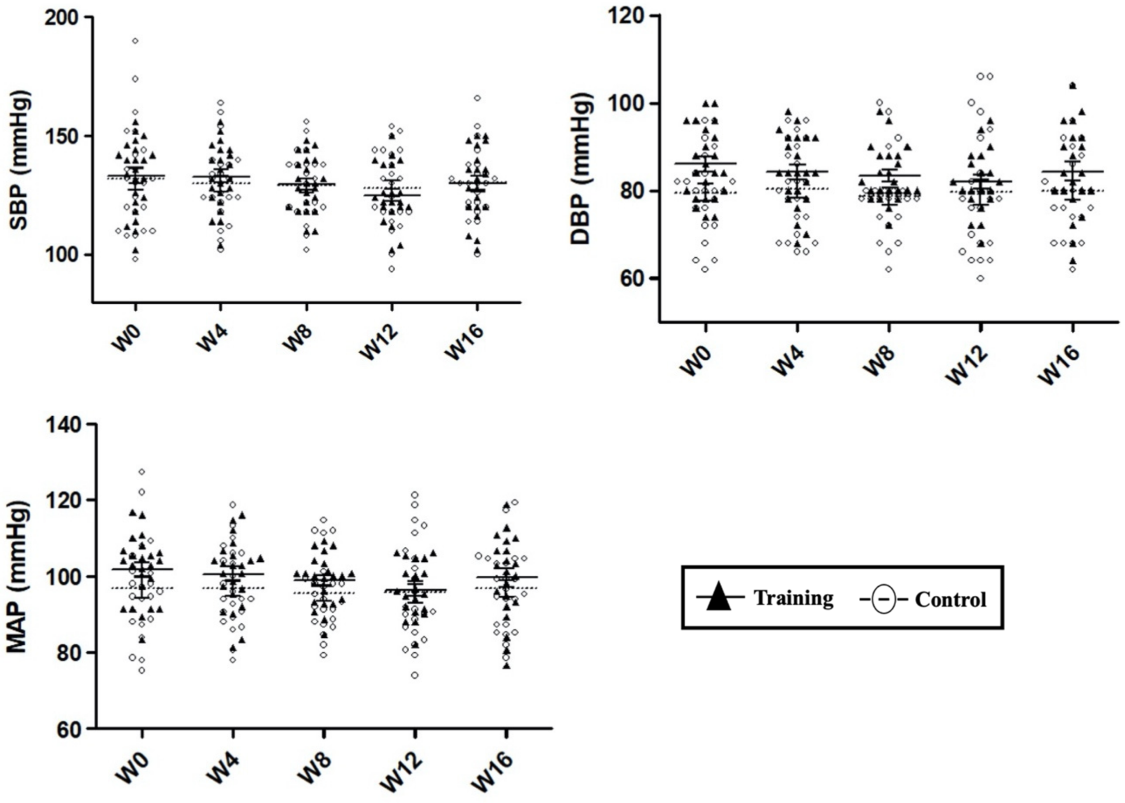

Figure S3b. Individual data, median and interquartile range for Cardiometabolic profile: Cardiovascular function.

Table S1. ANOVA 2 way for repeated measures at W0 and at W16.

	Within subjects Effects: group*time interaction	Pairwise comparison (P-Value)
Body composition		
Body Mass (kg)	F (1, 42) = 6.2, P = 0.017, η_p^2 = 0.13	0.231
BMI (kg/m^2)	F (1, 42) = 6.52, P = 0.014, ηp2 = 0.13	0.32
Fat mass (%) [NN]	F (1, 42) = 1.11, P = 0.298, ηp2 = 0.03	0.303
Fat free mass (%) [NN]	F (1, 42) = 1.09, P = 0.303, ηp2 = 0.03	0.299
VL thickness (cm)	F (1, 40) = 1.17, P = 0.286, ηp2 = 0.03	0.091
RF thickness (cm)	F (1, 41) = 3.66, P = 0.063, ηp2 = 0.08	0.557
Neck circumference (cm)	F (1, 42) = 3.51, P = 0.068, ηp2 = 0.08	0.018
Waist circumference (cm)	F (1, 42) = 24.25, P < 0.001, ηp2 = 0.37	0.011
Hip circumference (cm)	F (1, 42) = 8.15, P = 0.007, ηp2 = 0.16	0.502
Cardiovascular function		
SBP (mmHg)	F (1, 42) = 0.07, P = 0.797, ηp2 = 0	0.364
DBP (mmHg) [Log]	F (1, 42) = 0.51, P = 0.477, ηp2 = 0.01	0.676
MAP (mmHg)	F (1, 42) = 0.35, P = 0.56, ηp2 = 0.01	0.487
ABI [Log]	F (1, 41) = 3.95, P = 0.054, ηp2 = 0.09	0.025
SV (ml) P = 0.90	F (1, 36) = 0.31, P = 0.583, ηp2 = 0.01	0.347
CO (L/min) P = 0.90	F (1, 36) = 1.78, P = 0.191, ηp2 = 0.05	0.784
TPR (mmHg/min/l) [Log] P = 0.90	F (1, 36) = 3.22, P = 0.081, ηp2 = 0.08	0.647
IMT (mm)	F (1, 25) = 1.29, P = 0.267, ηp2 = 0.05	0.254
CC (%/10mmHg) [OR NN]	F (1, 18) = 5.14, P = 0.036, ηp2 = 0.22	0.665
Physical fitness.		
Isokinetic peak torque of knee extension (Kg) [Log]	F (1, 40) = 12.47, P = 0.001, ηp2 = 0.24	0.015
Isokinetic peak torque of knee flexion (Kg) [Log]	F (1, 40) = 2.88, P = 0.097, ηp2 = 0.07	P < 0.001
Isometric peak torque of knee extension (Kg) [Log]	F (1, 40) = 12.36, P = 0.001, ηp2 = 0.24	0.111
Isometric peak torque of knee flexion (Kg) [SqR]	F (1, 40) = 0.08, P = 0.774, ηp2 = 0	0.01
Rate of force development of knee extension [Log]	F (1, 40) = 16.5, P < 0.001, ηp2 = 0.29	0.259
Rate of force development of knee flexion [Log]	F (1, 39) = 1.31, P = 0.26, ηp2 = 0.03	0.447
VO_2max (ml/kg/min)	F (1, 39) = 11.23, P = 0.002, ηp2 = 0.22	P < 0.001
Maximal speed (km/h)	F (1, 39) = 42.03, P < 0.001, ηp2 = 0.52	P < 0.001
Balance (Berg Scale) [NN]	F (1, 40) = 0.48, P = 0.492, ηp2 = 0.01	0.072
Sit and reach (cm)	F (1, 40) = 0.29, P = 0.591, ηp2 = 0.01	0.876
Stand and sit (repetitions in 30s) [Log]	F (1, 40) = 2.75, P = 0.105, ηp2 = 0.06	P < 0.001
Handgrip strength (Kg) [Log]	F (1, 40) = 0.02, P = 0.875, ηp2 = 0	0.098
Timed up and go (s)	F (1, 40) = 0.23, P = 0.631, ηp2 = 0.01	P < 0.001
Gait speed (s)	F (1, 40) = 0.59, P = 0.447, ηp2 = 0.01	P < 0.001
Blood markers.		
Glycemic and Lipidic profile		
Blood glucose (mg/dl) [NN]	F (1, 40) = 0.51, P = 0.478, ηp2 = 0.01	0.118
Insulin (um/ml) [Log]	F (1, 36) = 1.86, P = 0.181, ηp2 = 0.05	0.73
HOMA-IR [NN]	F (1, 34) = 1.19, P = 0.284, ηp2 = 0.03	0.708
Triglycerides (mg/dl) [Log]	F (1, 40) = 2.47, P = 0.124, ηp2 = 0.06	0.78
Total cholesterol (mg/dl) [SqR]	F (1, 40) = 1.57, P = 0.218, ηp2 = 0.04	0.786

HDL (mg/dl) [Log]	F (1, 41) = 3.84, P = 0.057, ηp2 = 0.09	0.303
LDL (mg/dl)	F (1, 40) = 0.6, P = 0.444, ηp2 = 0.01	0.726
Inflammatory profile		
IL-6 (pg/ml) [NN]	F (1, 39) = 0.54, P = 0.469, ηp2 = 0.01	0.841
IL-1ra (pg/ml) [Log]	F (1, 39) = 0.07, P = 0.797, ηp2 = 0	0.297
TNF-α (pg/ml)	F (1, 39) = 0, P = 0.975, ηp2 = 0	0.498
PCR (mg/L) [Log]	F (1, 27) = 0.1, P = 0.755, ηp2 = 0	0.772
Adiponectin (ug/ml) [Log]	F (1, 39) = 0.78, P = 0.383, ηp2 = 0.02	0.791
Leptin (ng/ml) [Log]	F (1, 39) = 0.32, P = 0.574, ηp2 = 0.01	0.986
Oxidative stress profile		
Carbonils (nmol/mg) [Log]	F (1, 40) = 0.49, P = 0.488, ηp2 = 0.01	0.062
Hydrogen peroxide (μM) [NN]	F (1, 40) = 0.73, P = 0.398, ηp2 = 0.02	0.315
NADPH oxidase (ng/mg) [SqR]	F (1, 33) = 1.06, P = 0.31, ηp2 = 0.03	0.981
FRAP (mM Fe(ii) [Log]	F (1, 40) = 0.16, P = 0.689, ηp2 = 0	0.411
SOD (Usod/mg protein)	F (1, 40) = 0.22, P = 0.64, ηp2 = 0.01	0.575
Vasoactive substances		
ET-1 (pg/ml) [SqR]	F (1, 39) = 0.27, P = 0.608, ηp2 = 0.01	0.142
Nitrite (nmol/mg protein) [Log]	F (1, 40) = 0.15, P = 0.696, ηp2 = 0	0.802
Heart rate variability indexes: Time domain.		
FC (bpm)	F (1, 40) = 0.71, P = 0.406, ηp2 = 0.02	0.969
RRi (ms) [Log]	F (1, 40) = 0.62, P = 0.436, ηp2 = 0.02	0.956
SDNN (ms) [NN]	F (1, 40) = 0.33, P = 0.572, ηp2 = 0.01	0.658
RMSSD (ms) [Log]	F (1, 40) = 0.12, P = 0.733, ηp2 = 0	0.721
pNN50 (%) [Log]	F (1, 29) = 0.21, P = 0.65, ηp2 = 0.01	0.364
Heart rate variability indexes: Frequency domain		
HF (ms^2) [Log]	F (1, 40) = 0, P = 0.983, ηp2 = 0	0.67
LF (ms^2) [Log]	F (1, 40) = 0, P = 0.968, ηp2 = 0	0.927
TP [NN]	F (1, 40) = 0.18, P = 0.671, ηp2 = 0	0.954
LF/HF [Log]	F (1, 40) = 0.52, P = 0.475, ηp2 = 0.01	0.429
HF (nu)	F (1, 40) = 0.17, P = 0.683, ηp2 = 0	0.785
LF (nu)	F (1, 40) = 0.33, P = 0.571, ηp2 = 0.01	0.686
Heart rate variability indexes: Non-linear analysis.		
SD1 [Log]	F (1, 40) = 0.43, P = 0.516, ηp2 = 0.01	0.508
SD2 [NN]	F (1, 40) = 1.13, P = 0.294, ηp2 = 0.03	0.894

Legend: BMI: body mass index VL: vastus lateralis; RF: rectus femoris; SBP: systolic blood pressure; DBP: diastolic blood pressure; MAP: mean blood pressure; ABI: ankle-brachial index; SV: stroke volume; CO: cardiac output; TPR: total peripheral resistance; IMT: intima media thickness; CC: carotid compliance. [Log]: [SqR]: Analysis of square root transformed data; Analysis of log transformed data; [NN] Analysis of non-normally distributed data; [OR]: analysis with outliers removed; HDL: High density lipoprotein; LDL: Low density lipoprotein; IL-1ra: interleukin 1 receptor antagonist; IL-6: interleukin 6; TNF-α: Tumor necrosis factor alpha; CRP: C-reactive protein; TBARS: Thiobarbituric acid reactive substances; NASDH: nicotinamide adenine dinucleotide phosphate oxidase; FRAP: Ferric Reduction Ability Power; SOD: superoxide dismutase; ET-1: endothelin-1; HR: heart rate; RRi: R–R interval; SDNN: standard deviation of all normal R–R intervals; RMSSD: square root of the mean squared differences of successive R–R intervals; pNN50: Percentage of successive RR intervals that differ by more than 50 ms; HF: high frequency; LF: low frequency; VLF: very low frequency; TP: total power; LF/HF: ratio between the bands of low and high frequencies; SD1: Poincaré plot standard deviation perpendicular the line of identity; SD2: Poincaré plot standard deviation along the line of identity.
